# Estimating the Effectiveness of Health-Risk Communications with Propensity-Score Matching: Application to Arsenic Groundwater Contamination in Four US Locations

**DOI:** 10.1155/2014/783902

**Published:** 2014-09-30

**Authors:** Andrew J. Leidner

**Affiliations:** Department of Agricultural Economics, Texas A&M University, College Station, TX, USA

## Abstract

This paper provides a demonstration of propensity-score matching estimation methods to evaluate the effectiveness of health-risk communication efforts. This study develops a two-stage regression model to investigate household and respondent characteristics as they contribute to aversion behavior to reduce exposure to arsenic-contaminated groundwater. The aversion activity under study is a household-level point-of-use filtration device. Since the acquisition of arsenic contamination information and the engagement in an aversion activity may be codetermined, a two-stage propensity-score model is developed. In the first stage, the propensity for households to acquire arsenic contamination information is estimated. Then, the propensity scores are used to weight observations in a probit regression on the decision to avert the arsenic-related health risk. Of four potential sources of information, utility, media, friend, or others, information received from a friend appears to be the source of information most associated with aversion behavior. Other statistically significant covariates in the household's decision to avert contamination include reported household income, the presence of children in household, and region-level indicator variables. These findings are primarily illustrative and demonstrate the usefulness of propensity-score methods to estimate health-risk communication effectiveness. They may also be suggestive of areas for future research.

## 1. Introduction 

The provision of high quality drinking water is a priority for many regions of the globe [1–3]. In particular, arsenic contamination of drinking water supplies is a public health issue of growing importance. In some of the rural parts of Bangladesh, arsenic contamination has been an ongoing public health scourge [4], causing economic harm in the form of reduced supply of household labor [5]. Bangladeshis are exposed to arsenic by consuming water from wells which tap into contaminated aquifers [6]. In addition to Bangladesh, arsenic-contaminated aquifers exist in many parts of the globe, including China [7], Sri Lanka [8], Argentina [9], and several regions in the US [10]. In the US, the growing awareness and concern over arsenic contamination have prompted regional water leaders and water managers to investigate this issue and identify strategies that may be effective in reducing risks to public health [11–14]. The health-related harms associated with arsenic exposure include increased risks of a wide variety of diseases and outcomes [15], including skin lesions [16], cancers [17], and increased mortality rates [18]. Some of these health risks may be intensified when coupled with other known cancer-causing behaviors, such as cigarette smoking [19].

Contact and ingestion of arsenic-contaminated water can be avoided through public and/or community-level activities, such as enhancing water treatment processes at the point of distribution. These top-down, public-good efforts may require buy-in and implementation across regions, across levels of government, and may involve complex cost-sharing arrangements [20]. Health-risk aversion activities can also occur at the household level, where such activities may be the private response of local residents and families or parts of a larger publically funded campaign or project. Effective point-of-use treatment systems in the household for arsenic contamination can include reverse osmosis systems [14], ceramic filters specifically designed for arsenic removal [21], or even the purchase and consumption of arsenic-free bottled water [22]. This study investigates health-risk reduction and aversion activities at the household level in the context of several US arsenic-contaminated locations.

Previous research on the analysis of household water use as it relates to health and water quality issues can be divided into two categories, focusing on either valuations of water improvements or household behaviors. Valuation measurements have been estimated by eliciting a willingness to pay for improvements in water quality [23] or reductions in water-related health risks [24, 25]. The second category of studies that focus on behavior, specifically household-level decisions, has investigated the nature and content of informational messages [26, 27] or the particular health-risk circumstances and the types of environmental contaminants affecting the household's health-risk aversion decision [28]. The present study falls within the behavior-focused category and adds to this growing literature by investigating the associations of different sources of water-related health-risk information may have with household-level health-risk aversion behavior. This paper proposes a two-stage model to separate the effect of information from a household's propensity to acquire health-related information.

For water resource planners and managers, the optimal portfolio of water-related, health-risk reducing strategies is likely to contain a mixture of public and private actions. Theoretically, ignoring the private actions in such circumstances may result in unnecessary and excessive spending by the public sector. More practically, understanding the aversion behaviors of residents allows water planners and managers to implement more precise and effective public outreach programs to achieve a community's water-related public health objectives.

The objective of this study is to implement a propensity-score matching analysis and to gain a better understanding of the characteristics of water consumers who chose to engage in private health-risk reducing activities in response to knowledge about regional arsenic contamination. Supposing that, first, a household acquires water-related health-risk information and then decides whether or not to engage in an activity that averts some or all of the health risks, the effect of information at the household level on the decision to engage in a water-contamination aversion activity is modeled as a two-stage process. In the first stage, the household's propensity to acquire water-related health information is estimated. Using the propensity scores estimated in the first stage to weight observations, the second stage estimates the associations between a household's decision to implement a point-of-use filtration device and the household's relevant characteristics as well as the source of water-contamination information. This paper proceeds with a description of the methods and the results and concludes with a discussion of results.

## 2. Methods

The conceptual model subsection describes the concern for the endogenous determination of health-risk information and aversion behavior in a household. The description of data highlights some of the sample characteristics, study limitations, and assumptions that are required to map the components of the dataset with the components identified in the conceptual model. Beyond the intuitive appeal of the two-stage model, the empirical benefits of modeling the aversion decision as a two-stage process are demonstrated by comparing estimation results from the two-stage model with estimation results from a reduced form (or naïve) model, which does not attempt to control for the endogeneity of acquiring health-risk information and averting a health risk.

### 2.1. Conceptual Approach

The distribution of health-related risk information is one of the more readily available, implementable, and potentially less expensive tools in the toolkit held by planners and managers looking to affect desired public health objectives within a water supply and public health system. The extent to which this tool is effective depends on many factors. Two broad categories of such factors may include the means of the information being transmitted through the population and the responsiveness of the population to information. The responsiveness of the local population is likely to depend on a number of factors. Some of those factors may include familiarity with water treatment processes, health issues, the particular contaminant involved, or the level trust and believability in the information source held by local residents and business owners. Once a household is informed about a water-related health issue, conditional on the value of the information along with other household characteristics, the household decision-making agent or decision-making process chooses whether or not to engage in some form of health-risk aversion activity.

The objective of this study is to isolate the effect of information and, specifically, the source of information on the household's decision to avert. For this study, information is defined as self-reported prior knowledge of arsenic contamination in the area and aversion is defined as self-reported use of a water treatment device in the home. Conceptually, prior knowledge and the decision to avert are correlated with several location and household characteristics. The discovery of a more extensive level of arsenic contamination in a region is likely to induce greater public awareness as well as higher rates of aversion activity adoption. Households that are predisposed to be aware of public health issues may be more likely to encounter public health-related information in addition to being more likely to adopt practices that reduce household health risks.

**Figure 1:**
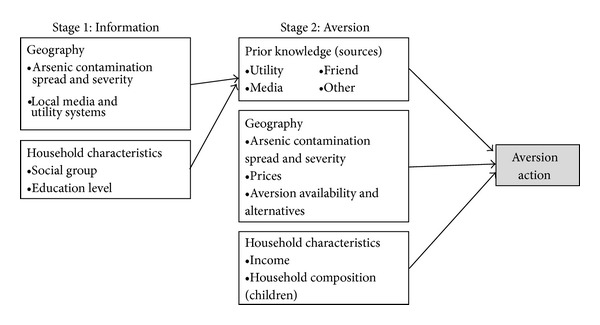
Conceptual model of two-stage decision process to engage in an aversion activity.

This study implements a two-stage model to represent this decision-making process (Figure 1). The first stage outcome is a household's possession of prior knowledge. The outcome of the second stage is the household's aversion decision, controlling for prior knowledge. The second stage controls for prior knowledge by using prior knowledge as a covariate in the aversion decision equation and also by weighting household observations by their estimated propensity to acquire prior knowledge. More details on the background and methodological description of using propensity scores and propensity-based regression weighting to estimate treatment effects can be found in the literature [29–31].

### 2.2. Data

The dataset used for this analysis has been used in several previous papers that address water consumption and health-risk behaviors in the context of arsenic-contaminated groundwater [19, 22, 32], where more extensive descriptions of the data and survey methods can be found. To summarize, survey respondents were randomly selected from within several arsenic contamination “hotspots” around the US. These areas include Albuquerque, New Mexico; Fernley, Nevada; Oklahoma City, Oklahoma; and Appleton and Outagamie County, Wisconsin. For this paper, some observations were dropped from the complete survey dataset because of missing values, mostly in the* INCOME* field. These characteristics of the data constitute sample biases, which should be considered when making inferences from the results of this study to a broader population.

Arsenic contamination aversion activities are captured by* POUFILTER*, an indicator for participation equal to one if the household uses any type of POU filtration device. Unfortunately, the specific type of POU device is not known. This means potentially that the POU device in service at a household may not be appropriate for the effective treatment and removal of arsenic. Respondents were also surveyed about their consumption of bottled water. Initial analysis showed no measurable association with bottled water drinking and information about arsenic contamination. As goods that are potentially consumed to reduce health risk, bottled water may be considered a substitute (or a complement) to POU filtration and as such may also be subject to the same codetermined endogeneity as household-level use of a POU device. Still, bottled water consumption has many other attributes. In particular, the effect on consumption from the convenience of bottled water may dwarf any perceived health-related benefits so as to make the health-related effect difficult or impossible to be measured in the current dataset. For this reason, bottled water is omitted from the presented analysis. In robustness checks, bottled water was considered as a covariate. In general, the relationship between bottled water consumption and use of a POU filter was negative, suggesting they may be substitutes in this sample. But this neither changed the direction nor substantially changed the statistical significance of the other estimates in the results of the two-stage model.

Like the aversion activity, prior knowledge is also captured by an indicator variable, descriptively named* PRIORKNOW,* and is equal to one if the respondent self-reports knowledge of the local arsenic contamination issue. The self-reported sources of any prior knowledge are captured by indicator variables as well:* FRIEND*,* UTILITY*,* MEDIA*, and* OTHERSOURCE*. These variables are not mutually exclusive. The variable* MEDIA *includes television, newspapers, and/or magazines. Most of the* OTHERSOURCE* responses are derived from open-ended responses. These responses varied between the following: indicating arsenic contamination information was “common knowledge,” the information was received from a real estate professional, the information was acquired due to the respondent's employment in a water supply-related business, or the information was received from a previous water-quality test at the household.

The other explanatory variables used in this study capture various characteristics of the households facing the arsenic aversion decision. The variables* AGE* and* SCHOOLING* are, respectively, the self-reported age in years of the respondent and the approximate highest grade level of schooling completed by the respondent. The variable* INCOME* is the approximate level of income for the household. The variable* HASCHILDREN* is an indicator variable equal to one if children are present in the household, where children are defined as persons who are under the age of 18.

Descriptive statistics are presented in Table 1. The descriptive statistics are presented for a pooled sample as well as two subsamples that represent, for the purposes of this study, the subsample of nonaverters and the subsample of averters. The nonaverters correspond to *POUFILTER* = 0 and the averters correspond to *POUFILTER* = 1. Just less than half (48.5%) of the households are averters. And, in the pooled sample, approximately 62% (or 166 respondents) reported knowing about the local arsenic contamination. Only 9% (or 25 respondents) of the pooled sample reported knowledge of the arsenic contamination and that a friend is a source of that knowledge.

Comparing averters to nonaverters, a larger percentage of averters report having obtained their prior knowledge from a* FRIEND* or* OTHERSOURCE*. Slightly fewer averting households reported obtaining prior knowledge from* UTILITY* or* MEDIA*. The averting subsample has higher average* INCOME* levels and a higher proportion of households with children present (*HASCHILDREN*). The two subsamples are fairly equal across* AGE* and* SCHOOLING*. The two subsamples also have different geographic distributions. Proportionally, more averting households are found in Nevada and Wisconsin, with proportionally fewer found in New Mexico and Oklahoma. The exact reasons for these differences in the geographic distribution of the subsamples are unknown but could be due to the heterogeneous influence and efforts of the local water supply system managers, health departments, the pervasiveness of arsenic-contamination-related knowledge, and the availability of goods and services that might be useful in aversion activities.

### 2.3. Empirical Approach

The statistical or empirical approach of this study investigates which of the four potential sources of prior knowledge has the greatest, or any measurable, effect on the household decision to avert arsenic-related health risks. Initially, all four sources of information are considered within two reduced form, or naïve, probit models. The first of these reduced form models, called the basic model, contains only the source of information as covariates. The second of these models, called the expanded model, contains information sources along with the full suite of available household characteristics.

The results from the reduced form models serve two purposes. First, in the basic reduced form specification, the entirety of the variation in* POUFILTER* is attributed to the source of information and an error term. Since no other covariates are included, relative to the other statistical models in the study, the basic reduced form model is the one most likely to measure some statistically significant effect on an information source, naively, due to confounding from one of many potentially omitted variables. Therefore, any statistically significant effect that is measured in the basic model requires additional investigation to determine if the effect is more likely to be causal or to be a measurement of correlated effects from other omitted covariates. The expanded reduced form model adds these additional covariates into the set of repressors to investigate if any of the effects measured in the basic model is reduced or reversed or made unmeasurable by the introduction of previously omitted variables. Controlling for these other covariates, any effects among the information sources that remain statistically significant in the expanded model suggest an information source that is more likely to reflect an actual causal association and more likely to produce measurable effects in later analyses.

The second purpose of the reduced form model is to underscore the usefulness of using the two-stage approach. The two-stage model is more conceptually intuitive and, as will be shown in the Results section, the two-stage model identifies more empirical effects which are in line with intuitive expectations about household behavior and averting health risks. The available data and variable names used in the empirical analysis are presented alongside the associated components of the conceptual model in Table 2.

Both equations of the two-stage model are estimated using a probit procedure. In the first stage, the outcome variable is* FRIEND*. The independent variables capture geographic and household characteristics by including geographic, state-level indicator variables (*NM*,* NV*, and* WI*), and household-level variables likely to be correlated with the household's social group and education level (*EDU*,* AGE*, and* INCOME*). After the propensity scores from the first stage estimation are recorded, a Gaussian kernel matching procedure is performed to identify the matched control sample. To achieve statistical balance between the matched control and treatment samples among the determinants of* FRIEND* propensity, quadratic terms of the household characteristics are also included as explanatory variables in the first stage. In robustness checks of the two-stage and the reduced form models, the logit procedure was used in place of the probit procedure, finding no qualitative differences in results. The discussion of results interprets the calculated marginal effects of the probit regressions.

The second stage outcome variable is* POUFILTER*. As with the first stage, the selection of independent variables reflects the best attempt to represent the second stage of the conceptual model with the available data (Table 2). Prior knowledge of the arsenic contamination event is captured by* FRIEND*. As in the first stage, the geographic characteristics are once again represented by group of state-level indicator variables. Household characteristics in the second stage are represented by the household-level variables* INCOME* and* HASCHILDREN*.

## 3. Results

The results are presented in three sections: the reduced form model, the first stage of the two-stage model, and the second stage.

### 3.1. Reduced Form Models

Marginal effects from the reduced form models are presented in Table 3. The statistical significance of* FRIEND* in both the basic and the expanded reduced form models motivates the later focus of this study on information from a* FRIEND*. In the basic reduced form model, the set of explanatory variables is limited to only the potential sources of information about arsenic contamination events (i.e., only* FRIEND, UTILITY, MEDIA,* and* OTHERSOURCE*). In so doing, relative to the other models presented in this study, the basic model attributes the most dependent variable variation to the information sources. Under these “generous” assumptions, statistically significant effects on* UTILITY* and* MEDIA* are not found. Statistically significant and positive effects on* FRIEND* and* OTHERSOURCE* are found. 

As the name implies, the expanded reduced form model expands the basic model by including the full set of potential cofactors. These results (Table 3) serve two purposes. First, they demonstrate the source of information is correlated with the other potential covariates (those covariates that are omitted from the basic model). In particular, the geographic indicator variables seem particularly influential, with statistically significant effects measured for* NV, NM, *and* WI*. With the alternative covariates included in the expanded model, the effect on* OTHERSOURCE* is no longer measurable. Interestingly, the effect on* FRIEND* remains statistically significant, albeit at 10% significance. The effect measured on* FRIEND* in the expanded reduced form model may be suggestive of a measurable causal relationship, which is more explicitly represented in the two-stage model. Finally, notice that none of the other household characteristics exhibit statistically significant effects. This raises concern that the reduced form model misspecifies the household-aversion decision, since past findings in the literature have indicated that both income [23] and the presence of children [33, 34] influence valuation of water supply improvements.

### 3.2. Stage 1: Information (Propensity-Score Estimation and Matching)

In the first stage, the propensity for being informed of arsenic contamination by a* FRIEND *is estimated.  Table 4 presents the mean values for the treated and matched control samples. The objective of the matching step (or first stage) is to obtain two samples (the treated and the matched control) that are balanced (or equal) in the values of the observed variables. No statistically significant differences are found among the treatment and matched control groups; however, the treated group only consists of 25 observations, which may provide insufficient power to identify a difference between the treatment and matched control samples. While their differences are not statistically significant, Table 4 shows that the two groups are not identical, particularly among the geographic indicator variables, where the treated group contains a higher proportion of observations from Wisconsin than from New Mexico, Nevada, and Oklahoma.

### 3.3. Stage 2: Aversion

Table 5 presents the results from the aversion stage with and without propensity-score weights. After adjustment for the propensity to be informed of arsenic contamination by a friend, the information from the friend exhibits a positive association with the probability of adopting an aversion practice. In addition to measuring a significant positive effect on* FRIEND*, the propensity-weighted model brings to light previously unmeasured associations. In contrast to the reduced form results of Table 3, the propensity-weighted model measures a significant positive effect on* HASCHILDREN *and* INCOME*. The effect of* HASCHILDREN *comports with previous findings in the literature [23, 33]. With other things being equal, households with children are more likely to perceive and respond to household health risks. Both the weighted and the nonweighted regressions measure a small effect for* INCOME*, suggesting that reductions in health risk behave like normal goods.

## 4. Discussion

Overall results presented in this study underscore the ability for households to privately pursue reductions to their water-related health risks. In particular, this research investigates whether or not any particular source of information seems more influential than another in motivating aversion behaviors. The primary finding of this study is that water-contamination information from a friend is found to have the strongest association with the adoption of an aversion activity, which in this case is defined as the implementation of a point-of-use filtration device in the household. Additional findings include positive and statistically significant associations between aversion and household income as well as aversion and the presence of children in the household. For the purposes practically affecting the aversion behavior of individuals exposed to arsenic contamination, the number of children and the income belonging to a household are not generally considered within the purview of environmental and public health groups. However, in the future, efforts to communicate health-risk information may be calibrated to take advantage of the most effective communication mediums and may be directed towards individuals, locations, or institutions with relatively extensive social networks.

The reduced form probit regressions served largely an exploratory role. Since the reduced form analysis pointed to knowledge from a friend as bearing the largest association with adoption of aversion practices, the subsequent analyses focused exclusively on prior knowledge from a friend. In robustness checks, the other sources of information were subjected to the same stage 1 and stage 2 analyses with no statistically significant result measured for their effects. While these results are generated from a propensity-score matching process that produced no statistically significant differences between the treated and matched control samples, the qualification remains that the number of treatment observations is relatively small, making any difference between the treated and match control groups relatively less likely to be statistically significant.

Another limitation worth discussion is the appropriateness of state-level indicator variables serving as proxy for the geographically correlated contributing factors in the 1st and 2nd stages. First, recall that respondents are surveyed from only one locale within each state; so, while these variables may be referred to as “state-level,” they actually capture a spatial scale closer to a city or county. In the 1st stage, geographic indicators proxy for the level of arsenic contamination across a region or a city and local utility and media networks across a region. In the 2nd stage, the state-level indicators proxy for the level of arsenic contamination, prices of aversion activities, and availability of aversion activities and alternatives. Of the two stages, the assumptions in 2nd stage seem less appropriate than in the 1st stage because, in particular, the availability and relative prices of aversion activities and aversion activity substitutes and complements seem more likely to be heterogeneous across households within a given region than the other variables (media and utility networks). Considered another way, local media and utility systems are more likely to be homogeneous across households within a given region; so, regional indicators are more appropriate.

More generally, these results are qualified by several contextual conditions, some of which were directly addressed in the modeling and others not directly addressed due to the unavailability of data. The dataset is not drawn from a statistically representative sample of any particular state, region, or the US; so, making inferences to larger populations is not warranted. Since the data is self-reported and from a survey, standard concerns over recall accuracy, reporting accuracy, and potential sample bias are applicable and therefore any inferences and generalizations to other populations are not recommended. Price data was not collected as part of the original survey and could not be reliably collected from other sources* ex post*. Presumably, these prices are somewhat consistent across given locations; so, while being not ideal, the price effect is assumed to be captured by the geographic indicator variables. Another potentially relevant socioeconomic factor that is similarly omitted includes the reliability and ease of access to retail locations for bottled water and POU devices. The use of POU devices as well as the consumption of bottled water is likely substitute goods (but could conceivably even be complement goods) for the purposes of avoiding health risks from arsenic contamination of groundwater. As such, a model that ignores the reciprocating relationship between two related goods is likely to be biased in some way, but for this study the data could not support a more rigorous approach. In a robustness check, an indicator for bottled water consumption is added as a covariate variable in the propensity-weighted stage-two equation. A significant effect is found for bottled water consumption, but this did not substantially change the effect found on* FRIEND*. In general, the small size of the dataset and particularly the small number of observations associated with the treatment variable* FRIEND* prohibit drawing stronger conclusions from any of the findings in this paper. This paper primarily provides a workable illustration of propensity-score matching for the purposes of estimating the relative effectiveness of health-risk communication methods.

## Figures and Tables

**Table 1:** Summary statistics for the unmatched data by sample type: pooled, nonaverters (*POUFILTER *= 0), and averters (*POUFILTER* = 1) among survey respondents in regions with arsenic contamination of groundwater.

Variable			Nonaverters		Averters	
	Pooled		*POUFILTER *= 0		*POUFILTER* = 1	
	Mean	Std. dev.	Mean	Std. dev.	Mean	Std. dev.
*POUFILTER *	0.485	0.501	0.000	0.000	1.000	0.000

*PRIORKNOW *	0.624	0.485	0.562	0.498	0.690	0.464
*FRIEND *	0.094	0.292	0.051	0.221	0.140	0.348
*UTILITY *	0.248	0.433	0.255	0.438	0.240	0.429
*MEDIA *	0.158	0.365	0.175	0.382	0.140	0.348
*OTHERSOURCE *	0.211	0.408	0.161	0.368	0.264	0.442

*AGE *	50.169	15.357	51.314	16.339	48.953	14.202
*SCHOOLING *	14.165	2.488	14.146	2.585	14.186	2.391
*INCOME *	68,224	37,100	64,708	36,387	71,957	37,625
*HASCHILDREN *	0.421	0.495	0.372	0.485	0.473	0.501

*NM *	0.143	0.351	0.226	0.420	0.054	0.227
*NV *	0.320	0.467	0.255	0.438	0.388	0.489
*OK *	0.241	0.428	0.314	0.466	0.163	0.371
*WI *	0.297	0.458	0.204	0.405	0.395	0.491

Number of observations	266		137		129	

**Table 2:** Components of the conceptual model and the associated variables used in the empirical model.

	Components of the conceptual model	Associated variables in the empirical model
Stage one	Prior knowledge	*PRIORKNOW *
	Utility	*UTILITY *
	Friend	*FRIEND *
	Media	*MEDIA *
	Other	*OTHER *
	Arsenic contamination spread and severity	*NM, NV, WI *
	Local media and utility systems	*NM, NV, WI *
	Social group	*INCOME*, *SCHOOLING, AGE *
	Education level	*INCOME*, *SCHOOLING, AGE *

Stage two	Aversion action	*POUFILTER *
	Prior knowledge	*PRIORKNOW *
	Utility	*UTILITY *
	Friend	*FRIEND *
	Media	*MEDIA *
	Other	*OTHER *
	Arsenic contamination spread and severity	*NM, NV, WI *
	Prices	*NM, NV, WI *
	Aversion availability and alternatives	*NM, NV, WI *
	Income	*INCOME *
	Household composition	*HASCHILDREN *

**Table 3:** Marginal effects from probit regressions on two reduced form models (basic and expanded) of point-of-use filter use among survey respondents in regions with arsenic contamination of groundwater.

	Basic reduced form		Expanded reduced form	
	Marg. eff.	S.E.	Marg. eff.	S.E.
*FR* *IE* *ND* ^a^	0.278∗∗∗	0.094	0.188∗	0.114
*UT* *IL* *IT* *Y* ^a^	0.033	0.074	−0.081	0.092
*ME* *DI* *A* ^a^	−0.027	0.085	−0.012	0.091
*OT* *HE* *RS* *OU* *RC* *E* ^a^	0.173∗∗	0.076	0.096	0.086

*EDU *			0.017	0.014
*AGE *			−0.001	0.003
*INCOME/10,000 *			0.015	0.009
*HA* *SC* *H* *IL* *D* *RE* *N* ^a^			0.038	0.083

*NM* ^a,b^			−0.161∗∗	0.082
*NV* ^a,b^			0.316∗∗∗	0.098
*WI* ^a,b^			0.280∗∗∗	0.091
Number of observations	266		266	
Adj-R^2^	0.032		0.123	

^a^Marginal effects of indicator variables calculated as a discrete change from 0 to 1.

^b^The referent category is *OK*.

^∗∗∗,∗∗,∗^refer to statistical significance at less than or equal to 0.01, 0.05, and 0.1.

**Table 4:** Balance table, the sample means for the treated and matched control samples among survey respondents in regions with arsenic contamination of groundwater.

Variable	Means		*P* value
	Treated	Control	
*EDU *	14.000	14.114	0.558
*AGE *	49.200	49.911	0.955
*INCOME/10,000 *	7.850	7.260	0.918
*NM *	0.080	0.107	0.980
*NV *	0.240	0.301	0.562
*OK *	0.080	0.137	0.647
*WI *	0.600	0.456	0.361

**Table 5:** Marginal effects from probit regressions on a model of point-of-use device adoption with observations weighted by the propensity to be informed by a friend about arsenic contamination of groundwater.

	Probit regression (with no propensity weights)		Probit regression with propensity weights	
	Marg. eff.	S.E.	Marg. eff.	S.E.
*FR* *IE* *ND* ^a^	0.184	0.112	0.207∗∗	0.098
*INCOME/10,000 *	0.017∗	0.009	0.024∗	0.013
*HA* *SC* *H* *IL* *D* *RE* *N* ^a^	0.053	0.067	0.273∗∗∗	0.098
*NM* ^a,b^	−0.147∗	0.082	−0.371∗∗∗	0.118
*NV* ^a,b^	0.285∗∗∗	0.082	0.293∗∗	0.146
*WI* ^a,b^	0.287∗∗∗	0.084	0.234	0.154
Number of observations	266		266	
Adj-*R* ^2^	0.113		0.198	

^a^Marginal effects of indicator variables calculated as a discrete change from 0 to 1.

^b^The referent category is *OK*.

^∗∗∗,∗∗,∗^refer to statistical significance at less than or equal to 0.01, 0.05, and 0.1.
